# Malnutrition among children under the age of five in the Democratic Republic of Congo (DRC): does geographic location matter?

**DOI:** 10.1186/1471-2458-11-261

**Published:** 2011-04-25

**Authors:** Ngianga-Bakwin Kandala, Tumwaka P Madungu, Jacques BO Emina, Kikhela PD Nzita, Francesco P Cappuccio

**Affiliations:** 1 University of Warwick, Warwick Medical School, Health Sciences Research Institute, Warwick Evidence, Gibbet Hill, CV4 7AL, Coventry, UK; 2 University of Botswana, Department of Population Studies, Gaborone, Private box 0075, Botswana; 3 Institut National de Statistique, Kinshasa, Republique Democratique du Congo; 4 African Populations and Health Research Center, Shelter Afrique Centre, Longonot Road, P.O.Box 10787, 00100 GP.O. Nairobi - Kenya; 5 Département des Sciences de la Population et du Développement, Faculté des Sciences Economiques, Université de Kinshasa, B.P. 176 Kinshasa XI, Republique Democratique du Congo; 6 University of Warwick, Warwick Medical School, Clinical Sciences Research Institute, Clifford Road Bridge, CV2 2DX, Coventry, UK

## Abstract

**Background:**

Although there are inequalities in child health and survival in the Democratic Republic of Congo (DRC), the influence of distal determinants such as geographic location on children's nutritional status is still unclear. We investigate the impact of geographic location on child nutritional status by mapping the residual net effect of malnutrition while accounting for important risk factors.

**Methods:**

We examine spatial variation in under-five malnutrition with flexible geo-additive semi-parametric mixed model while simultaneously controlling for spatial dependence and possibly nonlinear effects of covariates within a simultaneous, coherent regression framework based on Markov Chain Monte Carlo techniques. Individual data records were constructed for children. Each record represents a child and consists of nutritional status information and a list of covariates. For the 8,992 children born within the last five years before the survey, 3,663 children have information on anthropometric measures.

Our novel empirical approach is able to flexibly determine to what extent the substantial spatial pattern of malnutrition is driven by detectable factors such as socioeconomic factors and can be attributable to unmeasured factors such as conflicts, political, environmental and cultural factors.

**Results:**

Although childhood malnutrition was more pronounced in all provinces of the DRC, after accounting for the location's effects, geographic differences were significant: malnutrition was significantly higher in rural areas compared to urban centres and this difference persisted after multiple adjustments. The findings suggest that models of nutritional intervention must be carefully specified with regard to residential location.

**Conclusion:**

Childhood malnutrition is spatially structured and rates remain very high in the provinces that rely on the mining industry and comparable to the level seen in Eastern provinces under conflicts. Even in provinces such as Bas-Congo that produce foods, childhood malnutrition is higher probably because of the economic decision to sell more than the population consumes. Improving maternal and child nutritional status is a prerequisite for achieving MDG 4, to reduce child mortality rate in the DRC.

## Background

Malnutrition prevents children from reaching their full physical and mental potential. Health and physical consequences of prolonged states of malnourishment among children are: delay in their physical growth and motor development; lower intellectual quotient (IQ), greater behavioural problems and deficient social skills; susceptibility to contracting diseases [1,2]. Furthermore, child malnutrition is associated with approximately 60 percent of under-five mortality in Sub-Saharan Africa (SSA) countries [3].

The majority of studies on child nutritional status have described prevalence of malnutrition among under-five children and analyzed socioeconomic, demographic and cultural factors associated with child malnutrition in SSA [4-7]. However, little is known about the links between child's nutritional status and distal determinants including geographic location and the environment due to restricted methodologies.

Our study aims to investigate the impact of geographic location as a proxy for distal factors and their influences on nutritional status of children. The province of residence is taken as a modifiable variable which can help explain the variation of malnutrition among children between different provinces.

Four reasons justify the interest of this study: first, geographic location is an important modifier of known predictors of malnutrition and is associated with food security and accessibility, especially in the context of conflict affected country such as the DRC.

Second, through the use of our empirical methods we can investigate inequalities in childhood malnutrition by mapping the residual net effect of spatial pattern of malnutrition more flexibly than most previous work.

Third, the methods also allow us to investigate non-linear effects of some risk factors prior to and after controlling for the socioeconomic determinants. This enables us to determine to what extent the substantial spatial pattern of malnutrition is driven by socioeconomic factors or point to the influence of omitted variables with strong spatial structure or possibly conflicts, political or environmental and cultural factors or even epidemiological processes that account for this spatial structure.

Fourth, the worsening socioeconomic, cultural and political context of the DRC needs to be investigated. The DRC is one of the SSA countries characterised by extreme poverty, high incidence of childhood diseases, high mortality and poor infrastructure: 75 percent of people are malnourished [1]; hundreds of thousands of children have died due to malnutrition over the past 12 years [3]. Furthermore, the country continues to experience armed conflicts and political instability since 1990. However, regardless of the worsening socioeconomic, political and health situations little is known about inequalities in childhood malnutrition across socio-economic strata or provinces although preliminary reports from the existing national surveys highlight the problem of malnutrition among children.

## Background on study area

The DRC is the third largest country (by area: 2,344,858 km^2^) in Africa and with immense natural resources distributed across its 11 provinces. It is, with the population of more than 68 million, the eighteenth most populous nation in the world, and the fourth most populous nation in Africa, 62 percent of which are under the age of fifteen.

Poverty and vulnerability are the main characteristics of the Congolese population. First, the World Bank estimated that the DRC's per capita gross domestic product (GDP) in 1999 was 78 US Dollar. The GDP has since declined. External debt at the end of 2000 was 12.9 billion US$ which, according to the Word Bank, equals roughly 280 percent of the GDP and to 900 percent of the exports. The accumulated debt and severe economic decline are due to both recent war and decades of corruption and economic mismanagement [8,9].

Further, since 1996, the DRC has been hit by conflict, which has devastated and destabilized the country and claimed the lives of an estimated six million civilians [10]. People continue to live in crisis conditions in many parts of the country. The eastern provinces (Orientale, Katanga, North and South Kivu), and more recently the province of Equateur, are afflicted by violence.

The ongoing Congolese crisis has claimed more lives than any conflict since World War II [10], and it continues to be of concern to the international community. Despite many political agreements signed since the start of the conflict, there is little expectation and prospect for peace as lives of vulnerable groups such as women and children continue to be shattered as conflict re-emerged in the eastern part of the country and a new front of violence opened in the province of Equateur. These conflicts have continued to hinder the DRC's ability to drive development efforts forward, so the population continues to suffer the consequences. Compounding this situation is the lack of leadership, mismanagement, corruption, rapid deterioration of the socio-economic conditions and the fall of prices of mineral resources which the national economy rely on because of the global financial crisis, which resulted in a sharp drop in revenues and massive loss of employment. Little progress is made in the implementation of the Government's Priority Action Plan on agriculture as most resources are concentrated on the army. Programmes are urgently needed to improve food security and auto-dependence, which would thereby reduce the country's over-reliance on humanitarian interventions to address the long-lasting acute and chronic malnutrition the country, continues to face [11].

Thus, humanitarian needs in the country remain colossal. According to the Central Emergency Respond Fund report in 2008, conflict has generated up to 1.35 million internally displaced persons (IDPs) in only three provinces, corroding the coping mechanisms of millions of people. With the continuation of conflict and the actions of abusive armed groups have increased food prices, matched with the limited ability of productive areas to feed population centres due to logistic constraints have generated malnutrition rates of up to 20 percent in certain health zones [11].

Consequently, chronic malnutrition is a serious problem, affecting some 48 percent of children in the DRC [12].

Preliminary reports from three nutritional national surveys (the 1995 and 2001 Multiple Indicator Cluster Surveys (1995 and 2001 MICS) and the 2007 Demographic and Health Survey (2007 DRCDHS) show that nutritional situation in the DRC remains critical [12,13]. Specifically, nutritional status of children under the age of 5 indicated deterioration in terms of acute malnutrition (stunting, wasting and underweight). Stunting rate was respectively 34 percent in 1995, 31 percent in 2001, and 46 percent in 2007. The nutritional status of mothers is also critical: about 19 percent of them were suffering from low Body Mass Index (BMI) in 2007.

The ever worsening political climate in Eastern provinces, resulted in war since 1996, has created an unprecedented hardship on the population, especially on children as they are more prone than adults to suffer from nutritional deficiencies because of their physiologically less stable situation [8]. Very high malnutrition rates have been recorded in the war provinces because of insecurity. But even in peace areas untouched by the present conflict nearly half of the children are malnourished [14]. Malnutrition remains one of the main factors associated with the high childhood morbidity and mortality [15,16].

National estimates of malnutrition may conceal important intra-provincial differences due to diverse cultural norms that might affect nutritional practices and the impact of the ongoing conflict on food security. It is therefore, important to examine patterns of malnutrition at a more disaggregated province level.

We recognise that a province in a country such as the DRC is a large unit of observation, but the provinces' estimates are more informative compared to the use of national estimates of malnutrition.

There is no specific empirical study undertaken to investigate determinants of malnutrition among children in the DRC. We therefore investigate the impact of geographic location on childhood malnutrition while taking into account the effect of the important risk factors of malnutrition present in the DHS database that might confound or mediate the inequalities of the spatial patterns observed at the province-level in order to gain a good understanding of the extent of malnutrition in a post-conflict country. The results will enrich the current literature with recent data on malnutrition, making it more understandable and helping to establish more effective intervention policies to monitor and evaluate achievement of the Millennium Development Goals (MDGs) in countries devastated by conflict. The policy interventions that would not account for unobservable distal factors (such as conflicts, political, environment etc...) will not deliver the required outcomes and will prolong the vulnerability of children in the DRC.

## Geographic Location in the DRC DHS

By applying the spatial analysis to the disaggregated province-level, we are able to establish whether the spatial effects cross the boundaries between the provinces or are distinct, which would also give us a sense on the relative importance of policies versus geographic factors in causing malnutrition.

While the eastern provinces used to be the major food producers of the country, repeated looting of crops by armed groups and general insecurity over many years has undermined production.

In other parts of the country with better security conditions, crumbled infrastructure has significantly decreased the country's food production capacity. Households and major food importers maintain food reserves at a bare minimum because of the volatile political and economic environment, as well as the frequent threats of looting.

High prices have also hit the DRC hard. Food prices have increased by 52 percent in June 2009 compared to figures from May 2008 [1]. This is probably due to the lack of national policy for food production and the reliance of the DRC on food aid (the DRC relies 100% on aid). The financial and economic crisis has also affected mining activities. Acute malnutrition is at dangerously high levels in some parts of the DRC. Acute malnutrition is above the emergency threshold in the Kasaï provinces (centre). Even the worst affected parts of North Kivu do not have such high rates perhaps due to humanitarian interventions. Malaria, malnutrition, acute respiratory infections, tuberculosis, and diarrhea are the main causes of child mortality, according to the Ministry of Health. Deteriorating health conditions have allowed the resurgence of epidemics such as measles and typhoid fever.

As conflict continues to prevail in Province Orientale, South, North Kivu and Equateur, children are subject to starvation, and there is an increase in child mortality and morbidity. An almost total lack of basic health and social infrastructure has had a negative impact on child health.

## Methods

This study uses data from the 2007 DRC Demographic Health Survey (DRC-DHS), a national representative investigation on children's and women's health. The DRC-DHS data has comparable information on community and household characteristics as well as on nutrition and health of women aged 15-49 years and their children under-five years old at the time of the survey. The samples covered all regions, urban and rural areas. In total 9,000 households (3,690 in urban areas and 5,310 in rural areas) were sampled. All women between the ages of 15 to 49 living in these households were interviewed. Mother and under-five nutritional module covers a sub-sample of one household out of two from the 9,000 selected households. The data contains information on 9,995 women and 8,992 children under the age of 5. The DHS data is of good quality. However, the information provided by this survey is cross-sectional. The samples collected under the DHS survey is drawn together using stratified multistage sampling designs, often with over-sampling of smaller domains such as urban areas or certain regions of a country. In many instances, these data are subsequently analyzed using statistical software designed for simple randomly sampled data. Such analyses fail to take into account the impact of the underlying complex sampling design on regression parameter estimates. Consequently, conclusions drawn from these analyses may give misleading estimates on important health indicators on which public policies are based. Techniques that account for the survey design such as weighting, stratification, and hierarchical regression can be used. Furthermore, DHS data use cluster-sampling to draw upon women respondents via multistage sampling, where: at the first stage, a stratified sample of enumeration areas (villages/communities) is taken; at the second stage, a sample of households within the selected communities is taken; and finally, at the third stage, all women respondents (aged 15-49 years) in the sample households are included. Cluster sampling is a cost-saving measure, without the need to list all the households. However, statistically, it creates analytical problems in that observational units are not independent. Thus, statistical analyses that rely upon the assumption of independence are no longer valid. We focus on the hierarchical regression technique using Bayesian Geo-additive models to take into account the above mentioned issues.

## Nutritional status

According to the World Health Organization (WHO) [17], malnutrition has three commonly used comprehensive types named stunting, wasting and underweight measures by height for age, weight for height and weight for age indexes respectively.

Stunting or growth retardation or chronic protein-energy malnutrition (PEM) is deficiency for calories and protein available to the body tissues and it is inadequate intake of food over a long period of time, or persistent and recurrent ill-health. This height-for-age index (stunting) is less sensitive to temporary food shortages and thus seems to be considered as the most reliable indicator. Because studies have shown that wasting is volatile over seasons and periods of sickness and underweight shows seasonal weight recovery and being overweight for some children can also affect weight-for-age index [8].

Wasting or acute protein-energy malnutrition captures the failure to receive adequate nutrition during the period immediately before the survey, resulting from recent episodes of illness and diarrhoea in particular or from acute food shortage. Underweight status is a composite of the two preceding ones, and can be due to either chronic, acute malnutrition or PEM.

In the three surveys, nutritional status was assessed according to weight-for-age, weight-for-height and height-for-age using the US National Center for Health Statistics/WHO international reference tables and charts [17,18]. Wasting, stunting and underweight were defined as weight-for-height, height-for-age and weight-for- age of 2SD or more below the corresponding median of the reference population, respectively; while severe wasting, severe stunting was defined as 3SD or more below the same median, respectively.

We focused on stunted children (2 SD of height-for-age below the median value) as our covariates were better able to explain chronic than acute malnutrition. We used the Z-Score (in a standardized form) as a continuous variable to maximize the amount of information available in the data set.

It is worth mentioning that, because of the drawback of the international reference population in correctly capturing nutritional status of children around the world; recently a new reference standard has been generated from which Z-scores can be calculated. For the purpose of this paper, the use of the new reference standard would not change the qualitative results. A detailed discussion on the new reference standard can be found in [19].

**Figure 1:**
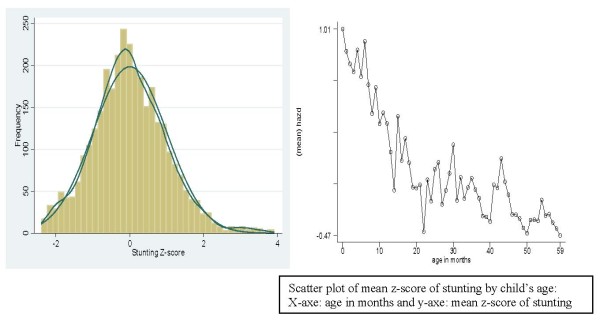
Histogram, kernel density of stunting (left) and mean standardized Z-score for stunting by child's age (right)

Figure 1 shows a histogram and kernel density estimates of the distribution of the Z-scores, together with a normal density, with mean and variance estimated from the sample. This gave us clear evidence that a Gaussian regression model is a reasonable choice for our inference for the dependent variable stunting.

## Correlates of Malnutrition

Child nutritional status is actually caused by multiple factors including, but not exclusively, those with illness, disease, and biological causes. A fuller understanding of illness and disease must include considerations of cultural, psychological, social and political factors present in the physical environment where the child lives. This premise has been expanded in many different areas such as medical, child psychology and sociology and now forms a fundamental part of a great deal of social science research and practice.

Mosley and Chen [20] in their study of the causes of death in children in developing and low income countries, placed risk factors within an analytical framework or including the interactions among socio-economic, cultural, environmental and biomedical factors. The framework focuses on the factors or determinants according to how direct the impact of the determinant was on the risk of death, i.e. the proximity of the risk posed to the children.

The Proximate factors include biological agents of disease e.g., microbes and vectors, and other elements which directly influence child's exposure to the agents of disease and ill health.

Distal factors include features of the wider socio-cultural, environmental and political context affecting both the child; his/her care givers e.g. public health policies and safety as well as cultural norms, environmental degradation which dictate how a family may respond to an illness.

These associations illustrate the vulnerability of children in any population who live in the environment where many of these determinants become unavailable or unstable.

Since we are interested in multiple causes of malnutrition, when modelling the determinants of malnutrition, we can distinguish between immediate, intermediate, and underlying determinants [3]. While malnutrition is always immediately related to either insufficient nutrient intake or the inability of the body to absorb nutrients (primarily due to illness), these are themselves caused by food security, care practises, and the health environment at the household level, which themselves are influenced by the socioeconomic and demographic situation of households, communities and public health policies [3,21,22]. Factors such as food security, care practises and health environment are a matter of public health policies. We refer to them as distal determinants of malnutrition.

In order to capture this complex chain of causation, various approaches have been taken each focusing on a particular level of causality. Studies [21,23] have estimated structural equations that address the interactions; Caputo, et al. [24] have used graphical chain models to assess the causal pathways, and other studies [5] have used multi-level modelling techniques. However, with the available data, it is not always clear how to separate intermediate from underlying determinants. For example, mother's education might be influencing care practises, an intermediate determinant, and the resources available to the household, an underlying determinant. On the other hand, child province of residence, a distal determinant, might influence food prices and security, intermediate determinants, and food availability, an underlying determinant.

Given these difficulties, our approach is to estimate models that mainly focus on factors that are mostly underlying determinants of malnutrition, although some might also be considered intermediate determinants and distal determinants. The most important covariate included in this analysis is the geographic location where the child lives that includes features of the wider socio-cultural and political context affecting both the child and his/her care givers. Other selected socio-demographics variables available in the data are grouped as individual child's characteristics, mother's characteristics, household economic level and community's characteristics. Regarding the covariates, we were guided by the previous literature on the subject and the conceptual framework outlined in [3].

Unfortunately, the surveys do not generate an income variable and we therefore rely on a household asset index as a proxy for the socio-economic status of the households which has been found to be quite reliable. Ownership of consumer items, such as a radio or car, as well as characteristics of the dwelling such as floor or roof type, toilet facilities and water source are items that measure poverty in these setting and the World Bank and others have used these items to generate an asset index, using Principal Components Analysis (PCA). We use the first principal component derived from the data to obtain the index for each household. We sort children by the asset index and establish cut-off values for percentiles of the population. We then refer to the bottom third as 'low socioeconomic status, the next third as 'medium socioeconomic status, the top third as 'high socioeconomic status' (see Table 1).

**Table 1:** Distribution of stunted* children by selected variables

Selected variables	Stunting: N = 3663		
	**N (%)**	**N (%)**	

	**Stunted:1607(43.9)**	**Not stunted: 2056(56.1)**	p-value**

**Sex of Child**			

Male	836(46.1)	979(53.9)	0.008

Female	771(41.7)	1077(58.3)	

**Age of child**			

0 years	185(23.1)	616(76.9)	< 0.001

1 year	367(46.5)	423(53.5)	

2 years	353(48.5)	375(51.5)	

3 years	333(49.4)	341(50.6)	

4 years	369(55.1)	301(44.9)	

**Sex of household's head**			

Male	1329(43.4)	1736(56.6)	0.16

Female	278(46.5)	320(53.5)	

**Place of residence**			

Urban	551(37.2)	929(62.8)	< 0.001

Rural	1056(48.4)	1127(51.6)	

**Place of delivery**			

Hospital	1094(41.8)	1526(58.2)	< 0.001

Other	479(49.1)	496(50.9)	

**Mother marital status**			

Married or living together	1459(43.8)	1873(56.2)	0.74

Single, divorced, widow...	148(44.7)	183(55.3)	

**Mother Education**			

None	429(49.8)	433(50.2)	< 0.001

Primary	766(47.0)	864(53.0)	

Secondary and high	412(35.2)	759(64.8)	

**Preceding birth interval**			

< 24 months	1005(43.5)	1305(56.5)	0.56

> 24 months	602(44.5)	751(55.5)	

**Asset Index**			

Poorest	402(49.8)	405(50.2)	< 0.001

Poorer	340(48)	369(52.0)	

Middle	347(45.5)	416(54.5)	

Richer	350(43.9)	448(56.1)	

Richest	168(28.7)	418(71.3)	

**Household size**			

Small (< 6 members)	581(44.8)	716(55.2)	0.65

Medium (6-10 members)	860(43.5)	1115(56.5)	

Large (> 10 members)	166(42.5)	225(57.5)	

**Provinces**			

Kinshasa	57(16.4)	290(83.6)	< 0.001

Bas-Congo	91(40.3)	135(59.7)	

Bandundu	140(42.4)	190(57.6)	

Equateur	108(36.7)	186(63.3)	

Orientale	88(35.3)	161(64.7)	

Nord Kivu	134(45.0)	164(55.0)	

Maniema	117(39.1)	182(60.9)	

Sud Kivu	130(46.1)	152(53.9)	

Katanga	142(44.4)	178(55.6)	

Kasai-Oriental	128(42.0)	177(58.0)	

Kasai-Occidental	137(46.1)	160(53.9)	

Mother's BMI***	21.5 (3.3)	21.9 (3.6)	0.007

* Data are presented as N and percentage.

**P-value for bivariate test (p-value at 0.05 levels)

***BMI is expressed as mean and standard deviation.

Among the underlying determinants of chronic malnutrition, we considered as a proxy measure of current or recent socioeconomic status (SES), the asset index, household size, the nutritional status of the mother (measured by her BMI), health knowledge and care practices measured by mother's education, mother's marital status, birth interval and place of delivery of children.

We also control for the sex of the child, urban rural location, and the age of child. Based on prior own work as well as other literature [17,22,23], we investigated a potentially non-linear pattern of effects of the mother's BMI as well as the age pattern on malnutrition. For illustration, the empirical distribution of the stunting Z-score by child's age is shown in Figures 1 (right). It is obvious that the effect of child's age on the mean Z-score of stunting is nonlinear. It will be difficult to model the possibly nonlinear effect of such covariates through a parametric functional form, which well justifies our use of a flexible semi-parametric model. Empirical distributions of all factors used in the analysis, are given in Table 1.

### Statistical analysis

Historically, variations in malnutrition prevalence has been related to household socio-economic factors because it determines the amount of resources (such as food, good sanitation, and health care) that are available to infants and neglected temporal and geographic gradients and other variations in risk, in order to generate hypotheses towards the cause of malnutrition.

We examine spatial variation in under-five malnutrition with flexible geo-additive semi-parametric mixed model while simultaneously controlling for spatial dependence and possibly nonlinear effects of covariates within a simultaneous, coherent regression framework. Individual data records were constructed for children. Each record represents a child and consists of nutritional status and a list of covariates. For the 8,992 children born within the last five years before the survey, 3,663 children have information on anthropometric measures. Because the predictor contains usual linear terms, nonlinear effects of metrical covariates and geographic effects in additive form, such models are also called geo-additive models. Kammann [25] proposed this type of models within an empirical Bayesian approach. Here, we apply a fully Bayesian approach as suggested in [26] which is based on Markov priors and uses Markov Chain Monte Carlo (MCMC) techniques for inference and model checking. For model choice, we routinely used the Deviance Information Criterion (DIC) developed in Spiegelhalter et al. [27], as a measure of fit and model complexity.

Geo-additive and geo-referenced disaggregated province level or site-specific analysis is a means of managing spatial and temporal variability of determinant of different types: distal, proximate and intermediate factors which are deemed to affect child nutritional status.

The aim of site-specific province analysis is to accelerate policy interventions, optimise inputs (unobserved factors such as distal ones: food security and prices policies, environmental etc...), improve child nutrition by taking into account the environmental impact and reduce the timescale to attain the Millennium Development Goals (MDGs). It is an approach that deals with multiple groups of factors input to improve child nutritional status in order to satisfy the actual needs of parts of the provinces rather than average needs of the whole country.

The analysis was carried out using version 0.9 of the BayesX software package [28], which permits Bayesian inference based on MCMC simulation techniques. The statistical significance of apparent associations between potential risk factors and stunting was explored in chi-square and Mann-Whitney *U*-tests, as appropriate. Multivariate analysis was used to evaluate the significance of the posterior mean determined for the fixed, non-linear effects and spatial effects. A *P*-value of < 0.05 was considered indicative of a statistically significant difference. We also run a sensitivity analysis for the choice of priors. Standard choices for the hyper-parameters are a = 1 and b = 0:005 or a = b = 0:001: Je?rey's Non-informative prior is closer to the later choice, and since practical experience shows that regression parameters depend on the choice of hyper-parameters, we have investigated in our application the sensitivity to this choice.

It would be beyond the scope of this paper to go into the details of estimation procedures. Please refer to Appendix 1 for a detailed explanation of the statistical methods. The method has also been discussed in more detail in [22].

## Results

Table 1 shows individual characteristics of the sample population prior to multiple adjustments of all factors that might confound or mediate the observed spatial variation within provinces on stunting.

Of the overall sample of 8,992 children, 41 percent (3,663) of the sample children had measurement on their height and weight to ascertain their nutritional status. Of those 50.8 percent was female and the overall prevalence of malnutrition (stunting) was 43.9 percent.

The prevalence of stunting was higher among boys compared to girls (46.1 versus 41.7 percent), has an inverse linear association with the age of the child (higher in the age groups ranging from 4 years, followed by 3 years, 2 years, 1 years but lower in the younger age (0 year): 55.1, 49.4, 48.5, 46.5 versus 23.1 percent), higher in rural areas compared with urban areas (48.4 versus 37.2 percent), higher among children born outside the hospital compared with their counterpart born in hospitals (49.1 versus 41.8 percent), linearly associated with maternal education (higher among children from non educated mother, followed by children from mothers with primary education but lower among children from mothers with secondary or higher education: 49.8, 47.0 versus 35.2 percent ), linearly associated with socio-economic status of the household (higher among children from the poorest household, followed by children from poor, middle or rich households but lower among children from richest households: 49.8, 48.0, 45.5, 43.9 versus 28.7 percent ), very high in Sud Kivu (46.1 percent) and Kasai Occidental (46.1 percent) provinces, followed by Nord Kivu (45.0 percent), Katanga (44.4 percent), Bandundu (42.4 percent), Kasai Oriental (42.0 percent), Bas Congo (40.3 percent), Maniema (39.1 percent), Equateur (36.7 percent), Orientale (35.3 percent) provinces, but lower in Kinshasa, the capital city (16.4 percent).

On the other hand, there were no statistically significant association observed between the prevalence of stunting and gender of the household's head, mother's marital status, preceding birth interval of the child, and household's size.

The geographical distribution of the crude prevalence of the standardized Z-scores for the response variable stunting by province display in Table 1 shows distinct spatial patterns. While in Kinshasa, Orientale and Equateur provinces, it appears that stunting was lower, there seem to be more areas of high stunting in North-Eastern of the DRC that is affected by conflict and the three provinces that relied heavily on local mineral mining (Katanga and the two Kasai). In addition to local small-area variability, there might also be an underlying smooth spatial component, which crosses provincial borders due to displacement of population during the conflicts, something we investigated below. The provincial prevalence shown in Table 1 also suggested that we should examine the spatial pattern of stunting at a more disaggregated province level as the national prevalence of 43.9 percent glossed over important intra-province differentials.

In the multivariate analysis the results for the fixed effects in Table 2 suggest that female children are slightly less stunted, as found in other studies [22,29]. In fact, the corresponding posterior mean, -0.12 for male, is negative and the 10% and 90% quintiles are both negative - indicating that the effect is statistically significant. Children living in rural areas are more stunted than their counterpart in urban areas. Maternal education rather than paternal education has a positive impact on children's nutritional status as well as household's socio-economic status. Children from low socioeconomic households were, as expected, more stunted than children from high income backgrounds.

**Table 2:** Provincial posterior mean for fixed effect parameters of malnourished children in DRC (DHS-2007)

Selected variables	adjusted	
	mean	[2.5% - 97.5% quintiles]

**Constant**	0.50	0.31; 0.71

**Sex of Child**		

Male	-0.12*	[-0.18; -0.06]

Female	0	reference

**Sex of household's head**		

Male	-0.02	[-0.12; 0.71]

Female	0	reference

**Place of residence**		

Urban	0	reference

Rural	-0.11*	[-0.20; -0.01]

**Mother marital status**		

Married or living together	0	reference

Single, divorced, widow...	-0.08	[-0.19; 0.03]

**Mother Education**		

None	-0.12*	[-0.23; -0.01]

Primary	-0.14	[-0.23; -0.06]

Secondary and high	0	reference

**Father Education**		

None	-0.08	[-0.21; 0.05]

Primary	-0.08	[-0.16; 0.002]

Secondary and high	0	reference

**Number of children under 5**		

Less than 2	0.07	[-0.002; 0.15]

More than 2	0	Reference

**Wealth Index**		

Poorest	-0.28*	[-0.44; -0.12]

Poorer	-0.28*	[-0.44; -0.14]

Middle	-0.27*	[-0.42; -0.13]

Richer	-0.30*	[-0.42; -0.18]

Richest	0	Reference

* A significant p-value for multivariate tests (p-value at 0.05 levels)

Model: adjusted model by controlling these variables: sex of child, place of residence, wealth index, mother education, province, household size...

**Figure 2:**
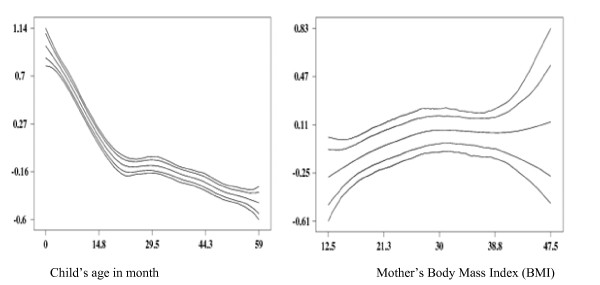
**Non-linear effects of and child's age (left) and mother's body mass index (right) on stunting**. Shown are posterior mean of stunting within the 80% and 95% credible interval

We also estimated the posterior mean of stunting and plotted it against child's age and mother's BMI. As hypothesised, Figure 2 shows that there is a bell shaped, non-linear relationship between the effects of child's age (left), mother's BMI (right) and stunting. Shown are the posterior means together with the 80% and 95% pointwise credible intervals. As found in other countries of SSA [22], these data show that the effect of mother's BMI on child's nutritional status to be in the form of an inverse U shape. While the inverse U looks nearly symmetric, the descending portion exhibits a much larger range in the credible region. This appears quite reasonable as obesity of the mother (possibly due to a poor quality diet) is likely to pose less of a risk for the nutritional status of the child as very low BMIs, which suggest acute undernutrition of the mother [22]. The Z-score is highest (and thus stunting lowest) at a BMI of around 30-35. The figure also shows that there are few women with high BMI (40 or higher) in the survey, but this is likely to represent an artefact of the small numbers sampled at this BMI range.

Figure 2 left shows the effect of the child's age on its nutritional status. As hypothesised and commonly suggested by the nutritional literature [22], we are able to discern the continuous worsening of the nutritional status up until about 20 months of age. This deterioration sets in right after birth and continues, more or less linearly, until 20 months. Such an immediate deterioration in nutritional status is not as expected as the literature typically suggests that the worsening is associated with weaning at around 4-6 months. One reason for this finding could be that, according to the surveys, most parents give their children liquids other than breast milk shortly after birth, which might contribute to infections at these early ages.

After 20 months, stunting stabilizes at a low level. Through reduced growth and the waning impact of infections, children are apparently able to reach a low-level equilibrium that allows their nutritional status to stabilize.

We also see a sudden improvement of the Z-score around 24 months of age. This is picking up the effect of a change in the data set that makes up the reference standard. Until 24 months, the currently used international reference standard is based on white children in the US of high socioeconomic status, while after 24 months; it is based on a representative sample of all US children [17]. Since the latter sample exhibits worse nutritional status, comparing the Congolese children to that sample leads to a sudden improvement of their nutritional status at 24 months [17,22].

This anomaly of the reference standard is one reason for the replacement of this reference population by a new reference standard from the WHO [19,29].

**Figure 3:**
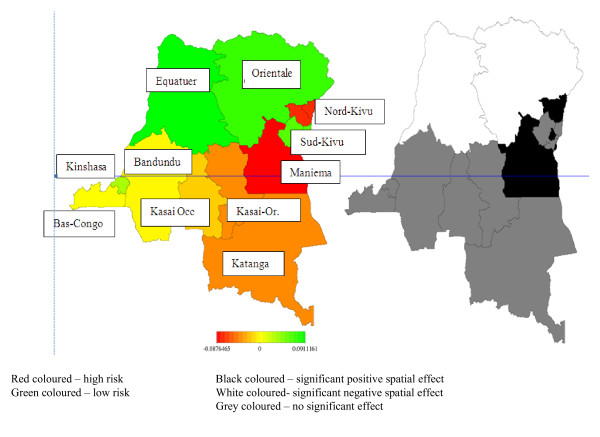
Total residual spatial effect of stunting (left) and posterior probabilities (right) of stunting for the full model

Figure 3 explores province specific net spatial effects of undernutrition. We report results of the model that includes the total residual spatial effects of the province (i.e. the sum of both the structured and unstructured spatial effects). The left panel of Figure 3 shows the total residual spatial effects of the province and the right panel of Figure 3 indicates the significance of the observed spatial effects in the form of a posterior probability map. The levels correspond to significantly negative (black colour), significantly positive (white colour) and insignificant (grey colour). Three important observations emerge. First, there is a strong north-south gradient in these provincial effects with a fairly sharp dividing line running through the centre of the country. Over and above the impact of the fixed effects, there appear to be negative influences of malnutrition in the south-east that are quite general and affect most of the provinces there. Given that the south-eastern provinces are all affected by the ongoing conflict than the rest of the country, it is likely that food security and price policies, environmental factors and associated conflict e.g. relying on food aids and, lack of public infrastructure, lack of farming due to conflicts are responsible for this pronounced regional pattern. Therefore, humanitarian assistance that the population mostly relies on in these conflict-affected provinces might have short-term impact on child nutritional status. Second, living in the capital Kinshasa and Sud-Kivu is associated with significantly better nutrition despite Sud Kivu being affected by the conflict and surrounded by provinces with negative effects (Nord Kivu and Maniema). Note that both rates of prevalence of stunting in Kinshasa and Sud-Kivu are above the emergency threshold of 15 percent. As in most developing countries, living in the capital provides access to nutrition and health care that is superior in ways that have not been captured adequately in the fixed effects. The advantage in nutritional status of children living in Sud- Kivu may be due to the fact that the province receives more food aid than any other province in the DRC. Many aids organizations are based in this province and there has been an influx of food aids in this province. Therefore, in the province of Sud-Kivu, children have probably more benefited from international food assistance. In other provinces that are affected by conflicts such as Nord-Kivu where many aid organizations are also based particularly in Goma and there has been an influx of food aid in this province, it is surprising that many children still suffer from severe malnutrition even though food is abundant where they live. One possible explanation is that the lack of food is due to the fear of cultivation in unsecured environment. Another possible explanation is that most children in these provinces live in displacement camps and the higher intensity of the conflict due to the predation of the abundant mineral resources in this area by armed groups [15].

In the two *Kasai*, one explanation for the high rate of stunting may be due to the fact that the first livelihood activities are wage labour and *mining *activities and few people are involved in agriculture. This explanation might also be true for for higher malnutrition observed in Katanga, which also relies on mining in addition to the impact of war.

To compare our province-specific nonlinear spatial effects with our simple fixed effects for provinces (Table 1), Figure 3 presents a map that shows those provincial effects. One can only distinguish three main provinces effects. Better nutritional status is found in the Orientale province and Equateur province as well as Kinshasa, worse nutritional status in the eastern provinces under conflicts and non significant effect for provinces in the south of the DRC. In contrast, the crude provincial fixed effects shown in Table 1 miss most of the findings we discussed above. In particular, the sharp North-South gradient present in the province analysis is clearly now visible as the three eastern provinces include provinces on both sides of that divide. Moreover, the positive effect of Kinshasa is simply averaged in with the Bas Congo and Bandundu provinces. Clearly, a lot is lost when relying on these crude estimates of modelling spatial effects.

## Discussion

The DHS data provides a consistent, large and national database that can be used to analyze patterns of malnutrition in the DRC. This study has shown the relationships between malnutrition and the geographic location as well as a number of other risk factors that could explain the site-specific variation at the province level.

Our results show that children's chronic malnutrition is highly prevalent in the entire country with rates largely above 40 percent. The DRC has a deficit of food and limited food productivity despite the country's enormous potential for agricultural production. Only the western part of the country is a net producer, in particular the province of Bas Congo.

Over the last ten years, there has been a significant decline of the production of almost all agricultural products. According to the World Food Programme (WFP), the production of cassava has decreased by 23 percent between 1992 and 2006; the production of plantain has decreased by 75 percent between 1990 and 2006. There has been an increase of the maize production (by 33 percent between 1990 and 2006) however in Maniema and North Kivu the production has decreased by 22, in Katanga by 12 percent.

The deterioration in food productivity is the result of many factors but can be attributed mainly to distal factors such as lack of implementation of national policy for food production, security and conflicts. The agricultural system is mainly subsistence-oriented. According to the WFP, more than 93 percent of households have access to land, however the majority cultivates less than 1 hectare, which does not allow for adequate production for sale or own consumption. Cultivation techniques are still very traditional and households lack farming tools. Few households have a plough or a tractor. Agricultural inputs, such as fertilizers are not available. Eight years after the launch of the government PMURR programme (Programme Multi sectoriel des Urgences pour la Reconstruction et la rehabilitation) to make fertilizers available to farmers, the programme has yet to make an impact on the agricultural sector. Also, the year 2010 was declared by the government as the agricultural year to push many reforms in the sector but the impact of such programmes is yet to be seen.

Seeds are often of low quality, and productivity is low. These are clearly areas where if there were a national policy, this could make a difference for the DRC. Also, in the Eastern provinces people do not cultivate due to the violence, in the provinces such as Katanga, the two Kasai and Orientale, the young generation has left the agricultural sector to work in the mining industries (gold, diamond and coltan). In the eastern provinces, only 18 percent of households own livestock. When they do, it is usually in small quantity. Goat is the main livestock owned [30].

The results of these rates are similar to the one of the other countries [30]. Likewise, the risk of stunting is higher in rural areas, among children from less educated mothers and living in poorer household after controlling for other variables in the model [7]. As in most countries of SSA, there is substantial spatial province difference in child nutritional status in the DRC. Kinshasa's population is essentially urban, the proportion of the most educated women is higher compared to other provinces and accessibility to health facilities and safe drinking water is better whereas rural children, or less educated mothers have difficult access to health facilities, and consume about half the calories daily than their urban counterparts [1].

The major finding of this study is that malnutrition rates remain very high in the provinces that rely on the mining industry (two Kasai and Katanga) comparable to the level seen in Eastern provinces under war. One possible explanation may be found in the nutritional behaviour of the population that do not give certain types of food to children on cultural grounds even though the food is nutritious and in the reliance of the population living in these provinces on artisanal mining industry and the neglect of agriculture. A survey on food security showed that Kasai occidental has the worst indicator on population availability for food. There is a real hunger problem in this province, because the population that lives in this province does not want to work in agriculture and it prefers to work in the traditional extraction of diamonds. Even in provinces such as Bas-Congo that produces foods, the population sells more than it consumes. The higher rate of malnutrition observed in the eastern provinces under war is not surprising; the lack of food is due to insecurity rather than their inability to produce food because these provinces are known as traditionally pastoral and agricultural provinces.

Another observation drawn from this paper (Table 1) is the gap in malnutrition rates between the province of Kinshasa and all other provinces. In fact, Kinshasa's stunting prevalence is very low compared with the national rate. But it is above the emergency threshold by humanitarian standard. In spite of the generalized state of poverty in the country, incomes are higher in Kinshasa; as a result, economically, the population of Kinshasa enjoys better access to food products. The presence of more educated mothers and their partners in Kinshasa, and the lowest rate of poorest people living there may permit better nutritional practices.

The strong evidence of statistically significant difference of malnutrition between socio economic groups mainly between poorest, poorer, middle and richer groups compared to the richest group confirms the reality that in the DRC affording food for the majority of the population is still a challenge [1]. According to the WFP, about 55 percent of households' expenditure is spent on food (only 40 percent in Bandundu). The main source of food is people's own production. The second source of food is the market, except for the two provinces of Kivu, where households rely first on the markets to access food.

Hence, in richer households, often children are well fed and cared for and provided with a safe and stimulating environment, through which they are more likely to survive, to have fewer diseases and illnesses, and to fully develop thinking, language, emotional and social skills [12]. But in poorer households, most children are affected by the resurgence of kwashiorkor - lack of proteins in the diet - although this remains controversial. This is certainly due to the increasing poverty among parents who cannot afford to buy proteins (groundnuts, beans, meat, fish, and milk) for their children. Findings are largely consistent with findings of others studies on malnutrition by socio economic status (SES) in SSA [7] and highlight that poorer children have a higher risk of becoming stunted than richer ones.

The gap observed on stunting prevalence between children from uneducated mothers or those whose mothers have a primary school level of education compared with those from mothers with secondary or high level of education remains high. In fact, education could make a difference by empowering mothers (decision on type of nutrition and/or use of preventive medicine). Similar results have been found in Cameroon [7] and in most developing countries [21]. Education could also help the mothers make informed nutritional decisions about cultural norms on certain types of food for children.

With reference to other variables, male children seem to be more exposed to the risk of malnutrition than female children. There is no obvious explanation for this gender difference but in Asia, for instance, gender's difference has been attributed to boys' preference over girls [29]. Also, older children are more prone to be exposed to anthropometric failure than their counterparts aged less than one. Mainly, older children are mixed breastfed, even not breastfed at times, while younger children may be protected by the mother's immune system at birth [22]. The risk could be also due to lack of foods in the households due to poverty or the lack of hygiene by mothers, when cooking children foods.

The direct causes of malnutrition are the lack of access to drinking water (in the DRC, it is estimated that more than two thirds of the population has no access to drinking water), morbidity (malaria, respiratory infections and diarrhoea) and poor food consumption [22,30]. Also, breast feeding practices are inadequate and according to the WFP, about 12 percent of the under 18 children are orphans. The prevalence changes significantly across the country, and it is higher in the East (more than 16 percent in province Orientale) [30].

## Conclusion

This study has been able to determine that in the DRC, childhood malnutrition is spatially structured and rates remain very high in the provinces that rely on the mining industry and comparable to the level seen in Eastern provinces under war. In war-affected provinces, we are able to determine that childhood malnutrition is higher probably because of the environmental impact caused by war because these provinces are known as traditionally pastoral and agricultural provinces. Furthermore, the massive influx of population especially from Rwanda, Uganda and Sudan fleeing conflicts has further exacerbated the food crisis. Food aids has helped but it is unsustainable. Even in provinces such as Bas-Congo that produce foods, childhood malnutrition is higher because of the economic decision to sell more than the population consumes.

In summary, in the DRC the improvement of the nutritional status of children would help avert child deaths from diarrhoea, pneumonia, malaria, HIV and measles. Consequently it would reduce neonatal mortality, helping achieve MDG 1, which main aim is to reduce poverty and hunger. There is an urgent need for national policies to improve the security of people and implement agricultural policies for auto-dependent agriculture (the DRC has the potential with plenty of land for agriculture). In other words, improving maternal and child nutrition is a prerequisite for achieving MDG 4, to reduce the child mortality rate. Also, nutritional programmes and policies that will try to reduce female illiteracy and provide basic infrastructures in rural areas in order to reduce gaps in health care between socio-economic groups are likely to succeed. The majority of the poorest household lives in rural areas and poorest children are more exposed to the risk of being malnourished. Hence, there is an urgent need to build programmes which aim to reduce poverty in both rural and urban areas, and which will take into account inequalities observed between provinces in the DRC.

## Competing interests

The authors declare that they have no competing interests.

## Authors' contributions

N-BK: Conception and design, literature review, data analysis and interpretation, drafting the article, critical revisions for important intellectual content and approval of final article for submission;

PTM: Literature review, interpretation of results, drafting the article, critical revisions for important intellectual content and approval of final article for submission;

JBOE: Interpretation of results and critical revisions for important intellectual content;

PDNK: Interpretation of results and critical revisions for important intellectual content;

FPC: Interpretation of results and critical revisions for important intellectual content; and all authors read and approved the final manuscript.

## Appendix

### Statistical analysis

Classical linear regression models of the form(1)

for observations (*y*_*i*_, *w*_*i*_), *i *= 1,....,*n*, on a response variable *y *and a vector *w *of covariates assume that the mean *E *(*y*_*i *_| *w*_*i*_) can be modeled through a *linear predictor w*_i_'*γ*. In our application to childhood under-nutrition and in many other regression situations, we are facing the following problems: First, for the *continuous covariates *in the data set, the assumption of a strictly linear effect on the response *y *may not be appropriate. In our study, such covariates are the child's age (*age*), the mother's age at birth (*mab*), and the mother's Body Mass Index (*BMI*). Generally, it will be difficult to model the possibly nonlinear effect of such covariates through a parametric functional form, which has to be *linear *in the parameters, prior to any data analysis.

Second, in addition to usual covariates, geographical small-area information was given in form of a location variable *s*, indicating the province, district or community where individuals or units in the sample size live or come from. In our study, this geographical information is given by the provinces of the DRC. Attempts to include such small-area information using province-specific dummy-variables would in our case entail more than 50 dummy-variables and using this approach we would not assess spatial inter-dependence. The latter problem cannot also be resolved through conventional multilevel modeling using uncorrelated random effects. It is reasonable to assume that areas close to each other are more similar than areas far apart, so that spatially correlated random effects are required.

To overcome these difficulties, we replace the strictly linear predictor through a *geo-additive predictor*, leading to the *geo-additive regression model*(2)

here, *f_1_,...,f_p _*are non-linear smooth effects of the metrical covariates, and *f_spat _*is the effect of the spatial covariate *s*_*i *_*∈ *{1,...,*S*} labelling the provinces in the DRC. Regression models with predictors as in (2) are sometimes referred to as geo-additive models. In a further step we may split up the spatial effect *f_spat _*into a spatially correlated (structured) and an uncorrelated (unstructured) effect: *f*_*spat*_*(s*_*i*_*) *= *f*_*str*_*(s*_*i*_*) *+ *f*_*unstr*_*(s*_*i*_*)*. The rationale is that a spatial effect is usually a surrogate of many unobserved influences, some of them may obey a strong spatial structure and others may be present only locally. The observation model (2) may be extended by including interaction *f(x)w *between a continuous covariate *x *and a binary component of *w*, say, leading to so called varying coefficient models, or by adding a nonlinear interaction *f_1,2 _(x_1_, x_2_) *of two continuous covariates.

In a Bayesian approach unknown functions *f_j _*and parameters γ as well as the variance parameter *σ^2 ^*are considered as random variables and have to be supplemented with appropriate prior assumptions. In the absence of any prior knowledge we assume independent diffuse priors *γ_j _α const, j = 1,...,r *for the parameters of fixed effects. Another common choice is highly dispersed Gaussian priors.

Several alternatives are available as smoothness priors for the unknown functions *f*_*j *_(*x*_*j*_), see [26]. We use Bayesian P(enalized) - Splines,. It is assumed that an unknown smooth function *f_j _(x_j_) *can be approximated by a polynomial spline of low degree. The usual choices are cubic splines, which are twice continuously differentiable piecewise cubic polynomials defined for a grid of k equally spaced knot *p *on the relevant interval [*a*,*b*] of the x-axis. Such a spline can be written in terms of a linear combination B-spline basis functions *B*_*m*_(*x*), i.e.(3)

These basis functions have finite support on four neighbouring intervals of the grid, and are zero elsewhere. A comparably small number of knots (usually between 10 and 40) is chosen to ensure enough flexibility in combination with a roughness penalty based on second order difference of adjacent B-spline coefficients to guarantee sufficient smoothness of the fitted curves. In our Bayesian approach this corresponds to second order random walks(4)

with Gaussian errors *u*_*m *_~ *N*(0,τ^2^). The variance parameter τ^2 ^controls the amount of smoothness, and is also estimated from the data. More details on Bayesian P-Splines can be found in [28]. Note that random walks are the special case of B-Splines of degree zero.

We now turn our attention to the spatial effects *f_str _*and *f_unstr_*. For the spatially correlated effect *f_str _*(s), s = 1, ... S, we choose Markov random field priors common in spatial statistics. These priors reflect spatial neighbourhood relationships. For geographical data one usually assumes that two sites or regions s and r are neighbours if they share a common boundary. Then a spatial extension of random walk models leads to the conditional, spatially autoregressive specification(5)

where *N_s _*is the number of adjacent regions, and *r ∈ ∂*_*s *_denotes that region r is a neighbour of region s. Thus the (conditional) mean of *f_str_(s) *is an average of function evaluations *f_str_(s) *of neighbouring regions. Again the variance *τ^2^*_*str *_controls the degree of smoothness.

For a spatially uncorrelated (unstructured) effect *f_unstr _*a common assumption is that the parameters *f_unstr_(s) *are i.i.d. Gaussian(6)

Variance or smoothness parameters *τ^2^*_*j*_, *j *= 1,...,*p*, *str*, *unstr*, are also considered as unknown and estimated simultaneously with corresponding unknown functions *f_j_*. Therefore, hyper-priors are assigned to them in a second stage of the hierarchy by highly dispersed inverse gamma distributions *p(τ*^*2*^_*j*_*) *~ *IG(a*_*j*_,*b*_*j*_*) *with known hyper-parameters *a_j _*and *b_j_*. For model choice, we routinely used the Deviance Information Criterion (DIC) developed in [27], as a measure of fit and model complexity.

## Pre-publication history

The pre-publication history for this paper can be accessed here:

http://www.biomedcentral.com/1471-2458/11/261/prepub
